# Genome mining: Prediction of lipopeptides and polyketides from *Bacillus* and related Firmicutes

**DOI:** 10.1016/j.csbj.2015.03.003

**Published:** 2015-03-24

**Authors:** Gajender Aleti, Angela Sessitsch, Günter Brader

**Affiliations:** AIT Austrian Institute of Technology GmbH, AIT, Health & Environment Department, Bioresources Unit, Konrad Lorenz Strasse 24, A-3430 Tulln, Austria

**Keywords:** Polyketides, Lipopeptides, Non-ribosomal protein synthetase, Genome mining, Structure prediction, *Paenibacillus*

## Abstract

*Bacillus* and related genera in the Bacillales within the Firmicutes harbor a variety of secondary metabolite gene clusters encoding polyketide synthases and non-ribosomal peptide synthetases responsible for remarkable diverse number of polyketides (PKs) and lipopeptides (LPs). These compounds may be utilized for medical and agricultural applications. Here, we summarize the knowledge on structural diversity and underlying gene clusters of LPs and PKs in the Bacillales. Moreover, we evaluate by using published prediction tools the potential metabolic capacity of these bacteria to produce type I PKs or LPs. The huge sequence repository of bacterial genomes and metagenomes provides the basis for such genome-mining to reveal the potential for novel structurally diverse secondary metabolites. The otherwise cumbersome task to isolate often unstable PKs and deduce their structure can be streamlined. Using web based prediction tools, we identified here several novel clusters of PKs and LPs from genomes deposited in the database. Our analysis suggests that a substantial fraction of predicted LPs and type I PKs are uncharacterized, and their functions remain to be studied. Known and predicted LPs and PKs occurred in the majority of the plant associated genera, predominantly in *Bacillus* and *Paenibacillus*. Surprisingly, many genera from other environments contain no or few of such compounds indicating the role of these secondary metabolites in plant-associated niches.

## 1 Introduction

Bacteria are known to produce structurally diverse secondary metabolites including aminoglycosides, polyketides (PKs) and several small proteinaceous and peptidal structures such as bacteriocins, oligopeptides and lipopeptides (LPs) [1–3]. A substantial number of these metabolites have been described for their bactericidal, immune suppression and tumor suppression properties and represent potentially valuable agents in medical and veterinary medical applications, but especially PKs and LPs play also essential roles for applications in agriculture. They are vital for bacterial activities in suppressing disease pressure in plants by antimicrobial activities and activating plant defense and are important for biofilm formation and root colonization of crop plants [4–8]. LPs and PKs encompass a variety of cyclic, linear and branched structures and are generated by complex enzymes known as non-ribosomal peptide synthetases (NRPS) and polyketide synthases (PKS), respectively [9,10]. NRPS and type I PKS share to a large extent similar modular architecture and are largely organized into modules containing multiple domains, allowing the repetitive incorporation of building blocks into larger resulting compounds [11]. However, for the biosynthesis of smaller compounds (e.g. some siderophores), non-modular NRPS have been reported [12]. Often NRPS and type I PKS enzymes work using a co-linearity code, so that the recruitment of amino acids (for NRPS) and carboxylic acids (for PKS) for the biosynthesis and final structure assembly is the same as the order of catalytic domains in the genome [13,14]. This feature and insight into the architecture of modules and domains of NRPS and PKS often facilitate prediction of compound structures based on genomic sequences [15,16]. Nevertheless, variations from this conventional organization have been described and include for instance module iteration and skipping in several biosynthetic processes [17].

In this review, we will focus on Bacillales, an order belonging to the phylum Firmicutes, as genera within this order represent a rich source for diverse secondary metabolite gene clusters. Based on a recent whole genome mining study, 31% of the Firmicutes are estimated to harbor NRPS and PKS secondary metabolite gene clusters. 70% of these encode NRPS and 30% hybrid NRPS/PKS or PKS [18]. The total percentage of Firmicutes producing secondary metabolites is certainly higher, also because genes responsible for many common secondary metabolite classes (e.g. many oligosaccharides) are not detected by widely used prediction tools such as antiSMASH[19,20]. The distribution of NRPS and PKS gene clusters within different orders of the Firmicutes is not uniform and *Bacillus* and *Paenibacillus* from the order Bacillales dominate this secondary metabolite gene clusters count. These two genera in particular are well noted for their capability to produce structurally diverse LPs and PKs [4,7], but the genome information from most other Bacillales members remains largely untapped.

Despite the fact that next generation sequencing technology has contributed to the ample availability of the whole genome sequence data and a number of analysis tools for metabolite prediction exist [19–23], yet little is accomplished to explore the sequence wealth to identify novel LPs and PKs in these genomes and to predict uncharacterized secondary metabolites. We briefly review current knowledge on well characterized LPs and PKs from the Bacillales and show which novel compounds can be anticipated based on published Bacillales genome data using genome mining study and secondary metabolite prediction tools. The questions addressed here are to review the structural and functional information and the underlying gene clusters of known type I PKs and LPs produced by Bacillales and to elucidate by genome mining potential products of uncharacterized gene clusters and the potential of producing yet unidentified secondary metabolites of these types in distinct taxonomic groups of the Bacillales.

### 1.1 *Bacillus* and *Paenibacillus* polyketides

Polyketides are generated from simpler building units by repeated decarboxylation and condensation cycles on PKS enzymes [24]. The PKS machinery comprises three core domains: the acyl transferase (AT), the acyl carrier protein (ACP) and the ketosynthase (KS). The AT domain is responsible for activation and transfer of a simpler building unit (malonyl coenzyme A) to the ACP domain. The KS domain catalyzes decarboxylation and condensation reaction between the two ACP linked malonates [25]. Other domains include ketoreductases (KR) which catalyze hydroxy group formation, dehydratases (DH) which form double bonds after water elimination, enoyl reductases (ER) which catalyzes reduction reaction of the double bonds and methyl transferases (MT) which introduce methyl groups and branching in the carbon backbone. A phosphopantetheinyl transferase (PPT) encoded by a *sfp* gene is essential for the activation of the ACP domains [26,27]. The arrangement and the order of the catalytic domains within PKS influence PKs biosynthesis leading to a remarkable diversity in the PKs production. The PKS enzymes can be broadly categorized into three types, depending on the architecture of catalytic domains [28]. Type I PKS enzymes contain modules organized in multiple catalytic domains within a single protein that carry out decarboxylation and condensation steps to generate PKs from the starter unit malonyl-CoA [11]. In the type II and type III PKS enzymes, catalytic domains are found in separate proteins [28]. A large group of bacterial PKs are produced by modular PKS I enzymes with iterative KS, ACP and modification domains. These type I PKS mostly lack AT domains within the clusters, malonyl-CoA is transfered by acyl transferases acting in trans [29]. A large number of PKS is often found in association with NRPS as hybrid enzymes type I PKS-NRPS [30].

Metabolites produced by *Bacillus amyloliquefaciens* and *Bacillus subtilis* represent a substantial part of the diversity of LPs and PKs from the genus *Bacillus* [31,32]. The majority of the plant growth promoting and biocontrol agents commercially available are produced by these two species [4]. They produce three types of polyene PKs comprising bacillaene, difficidin and macrolactin [26,32]. *B. amyloliquefaciens* FZB42 contains a genome size of 3918 kb, of which nearly 200 kb are devoted to the production of polyketides. These three giant PKs gene clusters were assigned unambiguously by a mutagenesis study, utilizing MALDI-TOF MS and LC-ESI MS techniques [26]. In the genus *Paenibacillus* two PKs have been described so far. The underlying genetic cluster remains to be unambiguously identified in the case of paenimacrolidin [33], while for the recently described paenilamicins from *Paenibacillus larvae* also the responsible gene clusters have been reported [34]. In the following we describe the five known types of PKS from *Bacillus* and *Paenibacillus* in more detail.

#### 1.1.1 Bacillaene

**Fig. 1:**
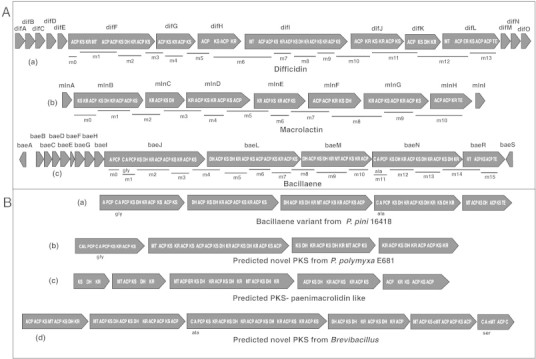
Architectures of type I polyketide synthases (PKS) showing similarities and dissimilarities in known and predicted PKs. Iterative domains: ACP, acyl carrier protein; PCP, peptidyl carrier protein; A, adenylation; KS, ketosynthase; DH, dehydratase; MT, methyl transferase; KR, ketoreductase; TE, thioesterase. Further details of domains are described in Table 1. Modules and recruited amino acids indicated below, gene names indicated above each illustration. (A) Gene clusters of the three types of well-known PKS from *B. amyloliquefaciens* FZB42: (a) difficidin, (b) macrolactin, (c) bacillaene. Modular regions of predicted PKS: (a) bacillaene variant from *P. pini* 16418; number and order of the domains differ from *B.amyloliquefaciens* FZB42 bacillaene, (b) novel PKS from *P. polymyxa* E681; an adenylation domain specifies glycine, (c) paenimacrolidine like PKS from P. durus DSM 1735, (d) novel PKS form *Brevibacillus brevis NBRC 100599;* adenylation domains specify ala and ser, also contains the methylation domains- oMT and nMT. These predicted PKS machinery in *Paenibacillus* may work without thioesterase.

**Fig. 2:**
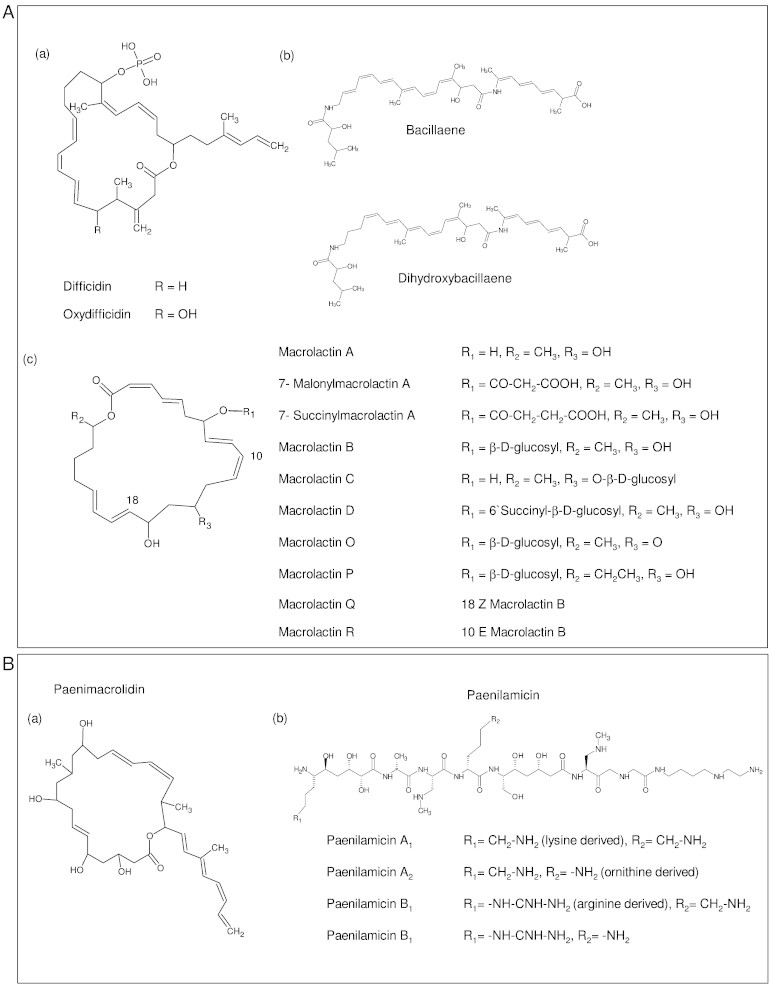
Chemical structures of polyketides of *Bacillus* and *Paenibacillus.* (A) Polyketides from *B. amyloliquefaciens* FZB42 (a, b, c) and *Bacillus* sp. AH159-1 (c): (a) difficidins, (b) bacillaenes and (c) macrolactins. Stereochemistry not shown. (B) Polyketides from *Paenibacillus*: (a) Paenimacrolidin from *Paenibacillus* sp. F6-B70. Stereochemistry unknown. (b) Paenilamicin from *P. larvae* DSM25430.

Bacillaene was first reported in the culture medium of *B. subtilis* strains 3610, and 55422 [35,36]. It has a linear structure comprising a conjugated hexaene (Fig. 2A) [35,36]. The biosynthesis of bacillaene has been described in *B. amyloliquefaciens* FZB42 and is encoded by a hybrid type I PKS-NRPS gene cluster called *bae* [26] (Fig. 1A). This cluster shares architectural characteristics with *pksX* of *B. subtilis* strain 168, presumably also encoding bacillaene [26]. The *bae* gene cluster contains five long open reading frames (ORFs) including *baeJ*, *baeL*, *baeM*, *baeN* and *baeR* [26]. The first and the second adenylation domains of *baeJ* are responsible for the incorporation of α-hydroxy-isocaproic acid and glycine, respectively. The third adenylation domain of *baeN* is involved in the incorporation of alanine [37]. Modules 4, 8 and 14 are splitted between adjacent genes (Fig. 1A). Three short ORFs found upstream of *baeJ* are *baeC, baeD, baeE*, encode for the three discrete AT domains that load malonyl-CoA [37]. Bacillaene and dihydrobacillaene are structural variants represented in this group of PKs [27,36] (Fig. 2A). Cell viable assays revealed that bacillaene selectively inhibits protein biosynthesis in prokaryotes, but not in eukaryotes, indicating a potential selective inhibition of other prokaryotes in their environment [35].

#### 1.1.2 Difficidin

Difficidin is known to be produced by *B. amyloliquefaciens* strains ATCC 39320 and ATCC 39374 (originally classified as *B. subtilis* in the original paper [38]), and is a highly unsaturated macrocyclic polyene comprising a 22 member carbon skeleton with a phosphate group rarely found in secondary metabolites. Oxydifficidin, a structural variant of difficidin has an additional hydroxyl group incorporated at position 5 [38] (Fig. 2A). Difficidin is encoded by the gene cluster dif with 14 open reading frames from *difA* to *difN* and *difY* (Fig. 1A). Difficidin and oxydifficidin biosynthesis deviates from the colinearity rule as a number of KR, DH and ER domains are absent within the gene cluster. So module 3 lacks the KR domain, module 4 and 5 two DH domains and modules 2 and 8 two ER domains, but these domains are found acting in trans. The contribution of the genes difJ and difK are unclear and their potential activities are not seen in the final product [26]. Difficidin shows antagonistic activity against broad range of bacteria [39]. Difficidin has been shown to be active against the phytopathogen *Erwinia amylovora* causing fire blight [31]. In *Escherichia coli* it has been demonstrated that difficidin is responsible for inhibiting protein biosynthesis [40].

#### 1.1.3 Macrolactin

Macrolactins have been isolated from *B. amyloliquefaciens* FZB42, the soil bacterium *Bacillus* sp. AH159-1 and from marine *Bacillus, Actinomadura* and uncharacterized species [41,42]. Most macrolactines are consisting of a 24 membered lactone ring with three diene moieties in the carbon backbone (Fig. 2A). The cyclic macrolactins are encoded in *B. amyloliquefaciens* FZB42 by the gene cluster *mln*, containing nine operons including *mlnA-I* [42] (Fig. 1A). The cluster contains 11 KS domains with malonate and acetate as the only used building units. Unlike in the bacillaene gene cluster, only one trans AT domain is found upstream of the *mlnA* gene. Similar to the *dif* gene cluster organization, *mln* shows an unusual splitting of the modules. Module 2 is splitted between *mlnB* and *mlnC* and a similar organization is seen for modules 5, 7, 8 and 10. A comparison of the order of the catalytic domains has shown that module 2 lacks the ER domain while modules 7 and 10 lack two DH domains. Like in *dif*, the activity of the missing domains can be accomplished by domains located in trans [42].

As the other *Bacillus* polyketides, macrolactins show antibacterial activity and might have the potential to be used in medical application [42]. In *in vitro* assays, they have also been shown to inhibit the proliferation of murine melanoma cancer cells and the replication of mammalian *Herpes simplex* virus and HIV in lymphoblast cells [43].

#### 1.1.4 Paenimacrolidin

Paenimacrolidin is a highly unstable macrocyclic lactone isolated from *Paenibacillus* sp. F6-B70 and comprises a 22 membered lactone ring with a triene in the carbon backbone [33] (Fig. 2B). Three out of four partial genes of the paenimacrolidin synthase showed high similarity to difficidin synthase of *B. amyloliquefaciens* and the structure of paenimacrolidin has similarities with difficidin, implying potential similarities in the biosynthesis and underlying genetic structures (Fig. 2A). Paenimacrolidin also exhibits antimicrobial activity against *Staphylococcus* with potential in clinical applications [3].

#### 1.1.5 Paenilamicin

Paenilamicins with antibacterial and antifungal activity have been isolated from *P. larvae* DSM25430, a honey bee pathogen [44]. Despite their activities these compounds do not seem to be involved in host killing, but rather in niche competition [34]. Based on gene activation studies the biosynthesis of paenilamicins has been assigned to the *pam* gene cluster (a complex NRPS/PKS hybrid gene cluster), and the structure (Fig. 2B) was elucidated using HPLC–ESI-MS, GC–MS, and NMR spectroscopy [34]. Different variants of paenilamicins are found due to variation in the first (lysine or arginine) and fourth (lysine or ornithine) recruited amino acid, but synthesis is performed by the very same enzyme complex encoded by *pam*. The non-ribosomal peptide synthetases 2, 3, 5, 6 and 7 encode alanine, N-methyl-diaminopropionic acid (mDap), serine, mDap and glycine, respectivly. Both PKS 1 and 2 mediate the formation of 2,3,5-trihydroxy pentanoic acid, which is then condensed to alanine. Finally, termination is achieved by nucleophilic cleavage by spermidine without involving thioesterase [34].

### 1.2 *Bacillus* and *Paenibacillus* lipopeptides

**Fig. 3:**
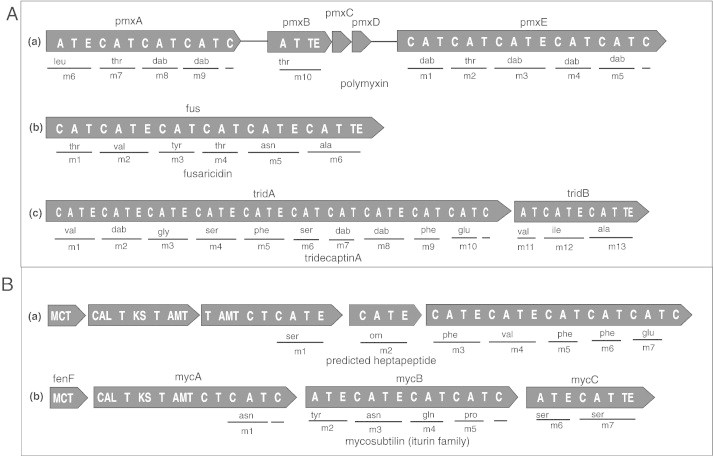
Organization of the non-ribosomal peptide synthetases (NRPS) encoding lipopeptides in *Paenibacillus* and *Bacillus.* Iterative domains: A, adenylation; T, thiolation; E, epimerization; MCT, malonyl-CoA transacylase; ACL, acyl-coA ligase; AMT, aminotransferase; dab, 2,4-diaminobutyric acid; orn, ornithine; KS, keto synthetase; TE, thioesterase. Further details of domains are described in Table 1. Modules and recruited amino acids indicated below, gene names indicated above each illustration. (A) Organization of the known NRPS (a) polymyxin A in *P. polymyxa* E681, (b) fusaricidin in *P. polymyxa* E681 and (c) tridecaptin A in *P. terrae* NRRL B-30644. (B) Organization of the predicted novel NRPS encoding (a) a heptapeptide in *P. polymyxa* E681; modular architecture is similar to the known Iturin but predicted amino acid composition is completely different and (b) organization of the known mycosubtilin operon [69], an iturin member from *B. subtilis* for comparison.

Lipopeptides from *Bacillus* and *Paenibacillus* have been described in a number of recent reviews [4,6,7,32,45,46]. These LPs are synthesized by non-ribosomal peptide synthetases (NRPS) [47]. NRPS comprise organized modules, each module containing catalytic domains: the adenylation (A) domain responsible for selection and monomer activation, the thiolation (T) domain for transfer of the adenylated monomer to a NRPS bound PPT, the condensation domain (C) for peptide bond formation and the thioesterase (TE) domain for release of the peptide monomer from NRPS. Also modification domains such as epimerization (E) domain catalyzing the isomerization of L- into D-amino acid monomers and methyl transferase (MT) are found. The starter condensation domain within the first module catalyzes the attachment of a fatty acid chain to the amino acid activated by the first adenylation domain [47] (Fig. 3). The gene clusters of the *Bacillus* LPs encoding the surfactin, fengycin, iturin and kurstakin families have been described and summarized in detail in a number of reviews [4,45,46].

**Fig. 4:**
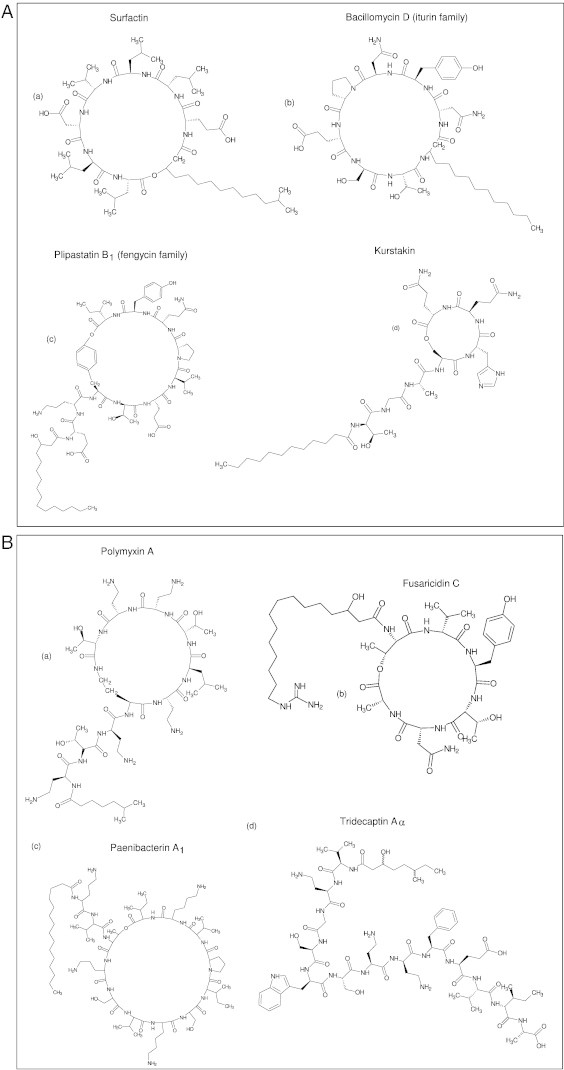
Chemical structures of lipopeptides from *Bacillus* and *Paenibacillus.* (A) Lipopeptides from *B. amyloliquefaciens* FZB42 (a,b,c): (a) surfactin, (b) bacillomycin (an iturin member), (c) plipastatin (a fengycin member) and (d) kurstakin from *B. thuringiensis kurstaki* HD-1. (B) Lipopeptides from *Paenibacillus*: (a) polymyxin A from *P. polymyxa* E681, (b) fusaricidin C from *P. polymyxa* E681, (c) paenibacterin from *Paenibacillus* sp. OSY-SE (d) tridecaptin from *P. terrae* NRRL B-30644.

Structurally, LPs consist of short oligopeptides (6–13 AA) with attached linear or branched fatty acids. For *Bacillus* and *Paenibacillus* linear and cyclic structures have been described (Fig. 4 shows examples of the variation) [7]. A large fraction of the *Paenibacillus* LPs are cyclic cationic LPs which contain the non-proteogenic amino acid 2,4-diaminobutyric acid (dab) contributing to the overall positive charge of the cationic lipopeptides. The polymyxins, octapeptins and polypeptins belong to this group enriched in dab (for review see [7]). The cationic lipopeptides have been reported as strong antibacterial agents against gram-negative bacteria and their mode of action is through permeabilization and disruption of the cell membrane [48,49]. Besides their clinical use as bactericidal agents, they have been shown to be active against plant pathogenic *Erwinia amylovora* and *Pectobacterium carotovorum*. [50]. The gene cluster responsible for synthesizing polymyxin synthetase has been described in plant growth promoting rhizobacteria such as *P. polymyxa* E681. The cluster encompasses five genes, of which *pmxA, pmxB* and *pmxE* encode the polymyxin synthetase, whereas *pmxD* and *pmxC* are involved in polymyxin transport [51] (Fig. 3A). Based on the amino acid substitutions at the positions 3, 6, 7 and 10, polymyxins are known to have variants (Fig. 4B). Octapeptins contain eight monomers and appear to be truncated polymyxins with cyclic heptapeptide structures in common. Like polymyxins they exhibit antibacterial activity against both gram-positive and gram-negative bacteria by acting on the membranes and are found in *Paenibacillus* spp. [52].

Polypeptins and pelgipeptins are cyclic nonapeptides isolated from *P. ehimensis* B7 and *P. elgii* B69, respectively. They are active against gram-positive and gram-negative bacteria, but also show antifungal activity against *Fusarium graminearum* and *Rhizoctonia solani* [53,54]. The gene cluster encoding pelgipeptin has been recently characterized in *P.elgii* B69 [55]. Other cyclic cationic LPs include gavaserin and paenibacterins. Gavaserin is isolated from *P. polymyxa* and speculated to contain a cyclic octapeptide structure [56]. Nevertheless, no structural data are available. Paenibacterins are known from *Paenibacillus* sp. OSY-SE and contain a tridecapeptide backbone (Fig. 4B). As the other cationic polypeptides they are active against gram-positive and gram-negative bacteria [57].

Cyclic noncationic lipopeptides from *Paenibacillus* comprise fusaricidins containing cyclic hexapeptide structure (Fig. 4B). They have been reported to exhibit strong antagonistic activity against *Fusarium oxysporum* [58]and induction of systemic resistance in red pepper plants against *Phytophthora* [59]. In addition, also a group of linear cationic LPs with different numbers of amino acids produced by *Paenibacillus* has been described. They include tridecaptins, with strong antimicrobial activity against gram-negative bacteria [60] (Fig. 4B). The gene cluster coding for tridecaptinA_α_ has been recently characterized from *P. terrae* NRRL B-30644 [61] (Fig. 3A). Cerexins are linear decapeptides, isolated from *B. cereus*, which display strong antimicrobial activity against gram-positive bacteria [62].

Most prominently*, B. amyloliquefaciens* and *B. subtilis* encompass gene clusters coding for cyclic LPs including surfactin, iturin, fengycin and kurstakin (46,63) (Fig. 4A). Several variants that differ in few amino acids have been reported within each family except for kurstakin. The LPs contain regularly variation in the fatty acid chain length and have linear, iso or aniso structural variations.

All surfactins contain cyclic heptapeptide structure, but differ in amino acid composition [64]. Known variants such as pumilacidin, lychenisin and surfactin represent this group and are remarkably confined to specific taxonomic groups [4]. Surfactins are vital for biofim formation and root colonization, but also exhibit a wide range of hemolytic, antimicrobial and antiviral activities, while fungicide activity has not been reported [65–68]. Surfactins are amphiphilic compounds, whose mode of activity seems mainly through membrane permeabilization and disruption [66].

All members of the iturin family have a cyclic heptapeptide structure, but differ from surfactins with distinct amino acid composition and cyclic closure of the lipopeptide structure by a beta-amino group of the fatty acid. Variants named bacillomycins, mycosubtilins, iturins and marihysins are noted [4,7,46]. They are mainly known for strong antifungal activity against several fungi [69–71]. Unlike surfactins their antibacterial activity is limited [72].

Fengycins and plipastatins are decapeptides which form a lactone ring structure between the C-terminus and a tyrosine at position three. They show remarkable antagonistic activity against filamentous fungi. The three LPs surfactin, iturin and fengycin may also act synergistically, enhancing their activities [73,74].

Kurstakins are another family of LPs isolated from *B. thuringiensis* strains and have been identified as phylogenetic markers for the species [75]. Kurstakins contain a lactone bond between Ser4 and the C-terminus of Gln7 and consequently form a cyclic tetrapeptide with a tetrapeptide side chain. They exhibit limited antifungal activity [63,75].

### 1.3 Genome mining tools for novel NRPS and PKS prediction

In order to discover novel secondary metabolites, several bioinformatics tools are available to perform genome mining. Some of the web based tools such as antiSMASH [20,21], NP.searcher [76] and NaPDoS [22] use hidden Markov models to identify NRPS and PKS in bacterial genomes. A more detailed prediction of the clusters is also possible through antiSMASH, which allows BLAST search on the predicted cluster to identify closest homologue in the database. antiSMASH allows the analysis of fragmented genomes and metagenomes making it a powerful prediction tool. Predicted peptides can be queried on NORINE database [77] containing more than 1000 non-ribosomal peptides to find similar structures [78]. Another useful prediction tool is the NRPS/PKS substrate predictor [23], which mainly focuses on the specificity of A domains (from NRPS) and AT domains (from PKS), which is useful to narrow the ambiguity of A domains specificity that occur in other prediction tools.

### 1.4 Prediction of lipopeptides and polyketides in published genome sets

In the following we evaluate the potential of type I PKs and LPs production based on genome mining and analysis, and show a clear potential for the discovery of several undiscovered variants and different structures. The next generation sequencing revolution of the last years have resulted and will result in a fast growing number of sequenced bacterial genomes and metagenomes. To evaluate the potential chemical space encoded in these genomes, the genome mining tools described above can facilitate the prediction of secondary metabolites, especially type I PKs and LPs. The cumbersome task, especially of various unstable PKs, to isolate and elucidate structures by NMR methods requiring milligram amounts can be pipelined by predicting the potential of novelty, also assisted by developments in mass spectrometry [79]. A limitation in prediction of PKs is that the colinearity rule common for LPs does not always apply. However, based on the predicted modular architecture and the number of core domains, it is still possible to predict the types of PKs and their variants as we show for Bacillales in the following (see Table 1 and Supplemental Table for an overview). A total of 160 published genomes the Bacillales were analyzed, of which 91 genomes contained metabolic clusters encoding LPs, type I PKs or both (57%). Intriguingly, a clear higher percentage, 85% of the 40 isolates, from rhizosphere and endophytes contained at least one of these metabolic clusters (Supplemental Table). However, the origin of almost a third of the isolates is unclear, making it difficult to foresee, if the higher incidence of these secondary metabolites in plant associated environments will also be seen when more genomes will be sequenced. A trend can be also seen phylogenetically with certain *Bacillus* spp. and *Paenibacillus* spp. as the taxa with the highest numbers of both type I PKs and LPs (Supplemental Fig.). How far also this observation just reflects a higher density of available genomes in these taxa than e.g. in *Salinibacillus* spp. remains to be seen.

In the following we evaluate the potential of type I PKs and LPs production based on genome mining and analysis, and show a clear potential for the discovery of several undiscovered variants and different structures. The next generation sequencing revolution of the last years have resulted and will result in a fast growing number of sequenced bacterial genomes and metagenomes. To evaluate the potential chemical space encoded in these genomes, the genome mining tools described above can facilitate the prediction of secondary metabolites, especially type I PKs and LPs. The cumbersome task, especially of various unstable PKs, to isolate and elucidate structures by NMR methods requiring milligram amounts can be pipelined by predicting the potential of novelty, also assisted by developments in mass spectrometry [79]. A limitation in prediction of PKs is that the colinearity rule common for LPs does not always apply. However, based on the predicted modular architecture and the number of core domains, it is still possible to predict the types of PKs and their variants as we show for Bacillales in the following (see Table 1 and Supplemental Table for an overview). A total of 160 published genomes the Bacillales were analyzed, of which 91 genomes contained metabolic clusters encoding LPs, type I PKs or both (57%). Intriguingly, a clear higher percentage, 85% of the 40 isolates, from rhizosphere and endophytes contained at least one of these metabolic clusters (Supplemental Table). However, the origin of almost a third of the isolates is unclear, making it difficult to foresee, if the higher incidence of these secondary metabolites in plant associated environments will also be seen when more genomes will be sequenced. A trend can be also seen phylogenetically with certain *Bacillus* spp. and *Paenibacillus* spp. as the taxa with the highest numbers of both type I PKs and LPs (Supplemental Fig.). How far also this observation just reflects a higher density of available genomes in these taxa than e.g. in *Salinibacillus* spp. remains to be seen.

Genome mining revealed the potential for known and novel LPs and PKs. Based on the prediction of the general architecture, undescribed, novel clusters can be identified (Supplemental Table, Table 1). Prediction of recruited substrates allows also the prediction of novel variants with same cluster architecture. Of course, even the same architecture and substrate prediction cannot exclude additional secondary modifications. These clusters were not considered as “novel” in the current analysis, but indicated as similar to described clusters in Table 1 and in the Supplemental Table. Especially in several *Paenibacillus* strains, we found a high potential for novel undescribed PKs and LPs variants of heptapeptides, nonapeptides, tridecaptins and decapeptides (truncated tridecaptins). Besides this, many *Paenibacillus* strains encompass known LPs such as polymyxins and fusaricidins and variants that differ in monomer composition (Table 1). We found also a novel fusaricidin variant in *P. massiliensis* DSM 16942 differing at the 4th position substituted by serine, which is believed to be highly specific for allo-threonine.

Genome mining revealed the potential for known and novel LPs and PKs. Based on the prediction of the general architecture, undescribed, novel clusters can be identified (Supplemental Table, Table 1). Prediction of recruited substrates allows also the prediction of novel variants with same cluster architecture. Of course, even the same architecture and substrate prediction cannot exclude additional secondary modifications. These clusters were not considered as “novel” in the current analysis, but indicated as similar to described clusters in Table 1 and in the Supplemental Table. Especially in several *Paenibacillus* strains, we found a high potential for novel undescribed PKs and LPs variants of heptapeptides, nonapeptides, tridecaptins and decapeptides (truncated tridecaptins). Besides this, many *Paenibacillus* strains encompass known LPs such as polymyxins and fusaricidins and variants that differ in monomer composition (Table 1). We found also a novel fusaricidin variant in *P. massiliensis* DSM 16942 differing at the 4th position substituted by serine, which is believed to be highly specific for allo-threonine.

Predicted heptapeptides from *Paenibacillus* strains have a modular architecture similar to iturin (Fig. 3B). Monomers of the peptide backbone in these heptapeptides are however completely different from the known iturin members. The genes in the heptapeptide operon of *P. polymyxa* E681 show up to 46% identity to bacillomycin D, an iturin member of *B. amyloliquefaciens* FZB42. Therefore, we hypothesize that these may belong to a novel class of iturins. Also, such heptapeptide variants with different peptide composition were found in other *Paenibacillus* strains such as *P. polymyxa* CR1, SC2, and *Paenibacillus* sp. HGH0039, *P. mucilaginosus* 3016 and *P. fonticola* DSM 21315. Moreover, we found an undescribed nonapeptide and its variants in *P. mucilaginosus* 3016, *P. elgii* B69 and *P. terrae* HPL-003. We discovered tridecaptin variants in *P. polymyxa* strains including E681, SQR21 and ATCC 842 (Table 1). In addition, we predicted decapeptides containing ten monomers, but with similar composition to tridecaptins. These seem to be truncated tridecaptins and therefore undescribed potential LPs of the *P. polymyxa* strains SQR21, M1 and SC2. We also identified a novel paenibacterin variant in *P. taiwanensis* DSM 18679 and *P. alvei* DSM 29 with four different amino acids to described metabolites of *Paenibacillus* sp. OSY-SE (Fig. 3B).

The majority of the *Bacillus* species that harbor lipopeptide gene clusters from the three families comprising surfactin, iturin and fengycin are *B. amyloliquefaciens*, *B. atrophaeus* and *B. subtilis*. Moreover, LPs (surfactins and fengycins) are predicted for *B. licheniformis*, *B. mojavensis* and *B. pumilus* with known metabolic potential but also for strains so far not characterized for their potential and less well investigated species such as *Salinibacillus aidingensis* (Table 1, Supplemental Table). The fourth family kurstakin is confined to *B. thuringiensis* strains. A kurstakin variant is found in *B. thuringiensis serovar kurstaki* HD73 with altered amino acid composition in position 2 and 5. The D and L forms of the monomers in a lipopeptide can also be predicted depending on presence and absence of the epimerization domains [80]. For instance, many *B. subtilis* encode plipastatin B, a member of fengycin family. Although plipastatin B and fengycin B are fengycin members and share identical monomers in the backbone, they differ in L-Tyr and D-Tyr, respectively, as also the chirality in monomers can be predicted with prediction tools. Altogether, it can be noted that the so far collected genome information confirms well known LPs for a number of *Bacillus* and *Paenibacillus* strains, but also shows a clear potential to produce a number of novel lipopeptides, especially in the genus *Paenibacillus*. A large number of strains from other genera of the Bacillales seem to lack the potential to produce LPs and PKs type 1 (Supplemental Table). However, it cannot be excluded that draft genomes may hinder the prediction of LPs and PKs (discussed below) if larger gaps within the published genomes exist.

The majority of the *Bacillus* species that harbor lipopeptide gene clusters from the three families comprising surfactin, iturin and fengycin are *B. amyloliquefaciens*, *B. atrophaeus* and *B. subtilis*. Moreover, LPs (surfactins and fengycins) are predicted for *B. licheniformis*, *B. mojavensis* and *B. pumilus* with known metabolic potential but also for strains so far not characterized for their potential and less well investigated species such as *Salinibacillus aidingensis* (Table 1, Supplemental Table). The fourth family kurstakin is confined to *B. thuringiensis* strains. A kurstakin variant is found in *B. thuringiensis serovar kurstaki* HD73 with altered amino acid composition in position 2 and 5. The D and L forms of the monomers in a lipopeptide can also be predicted depending on presence and absence of the epimerization domains [80]. For instance, many *B. subtilis* encode plipastatin B, a member of fengycin family. Although plipastatin B and fengycin B are fengycin members and share identical monomers in the backbone, they differ in L-Tyr and D-Tyr, respectively, as also the chirality in monomers can be predicted with prediction tools. Altogether, it can be noted that the so far collected genome information confirms well known LPs for a number of *Bacillus* and *Paenibacillus* strains, but also shows a clear potential to produce a number of novel lipopeptides, especially in the genus *Paenibacillus*. A large number of strains from other genera of the Bacillales seem to lack the potential to produce LPs and PKs type 1 (Supplemental Table). However, it cannot be excluded that draft genomes may hinder the prediction of LPs and PKs (discussed below) if larger gaps within the published genomes exist.

For the defined structure of the polyketide paenimacrolidin from *Paenibacillus* sp. F6-B70, the biosynthetic gene cluster is not characterized. Based on partial 16S rRNA gene analysis of *Paenibacillus* sp. F6-B70 it has been shown to be closely related to *P. elgii* and *P. ehimensis* [33]. We predicted a novel polyketide gene cluster that is similar in *P. durus* DSM1735, *P. elgii* and *P. ehimensis* (Fig. 1B). The partial paenimacrolidin synthase genes from *Paenibacillus* sp. F6-B70, have high similarity with part of *P. durus* genome. Furthermore, by examining the structure of paenimacrolidin using prediction tools, we speculate that a gene cluster with similarity to the difficidin cluster of *B. amyloliquefaciens* FZB42 may be responsible for the production of paenimacrolidin or a related PKS in these species (Table 1).

A number of very likely novel PKs with gene cluster architecture similar to bacillaene (Fig. 1B) are found in the *P. polymyxa* strains E681, SQR21, in *P. pini* JCM 16418 and in *Brevibacillus brevis* NBRC 100599 (Table 1). Intriguingly, in *P. polymyxa* strains, only one adenylation domain specifying glycine was found, instead of glycine and alanine as described in the bacillaene producer *B. amyloliquefaciens* (Table 1). PKS modules from *P. polymyxa* E681 shared up to 43% nucleotide sequence identity with *baeN* of *B*. *amyloliquefaciens*. Also for this polyketide, we identified variants that differ in number of the catalytic domains KS, DH, cMT and KR. In other *P. polymyxa* strains such as ATCC 842, M1 and SC2 a similar PKS cluster can be found with one DH domain less (Supplemental Table). In *P. pini,* the first adenylation domain specifies glycine like in bacillaene, while the second adenylation domain specifies serine instead of alanine. In *B. brevis,* the first adenylation domain specifies alanine and the second adenylation domain specifies serine. Besides it contains special methylation domains such as oMT and nMT that are not found in other polyketide clusters, clearly pointing to an uncharacterized PKs encoded in this genome (Fig. 1B).

A number of very likely novel PKs with gene cluster architecture similar to bacillaene (Fig. 1B) are found in the *P. polymyxa* strains E681, SQR21, in *P. pini* JCM 16418 and in *Brevibacillus brevis* NBRC 100599 (Table 1). Intriguingly, in *P. polymyxa* strains, only one adenylation domain specifying glycine was found, instead of glycine and alanine as described in the bacillaene producer *B. amyloliquefaciens* (Table 1). PKS modules from *P. polymyxa* E681 shared up to 43% nucleotide sequence identity with *baeN* of *B*. *amyloliquefaciens*. Also for this polyketide, we identified variants that differ in number of the catalytic domains KS, DH, cMT and KR. In other *P. polymyxa* strains such as ATCC 842, M1 and SC2 a similar PKS cluster can be found with one DH domain less (Supplemental Table). In *P. pini,* the first adenylation domain specifies glycine like in bacillaene, while the second adenylation domain specifies serine instead of alanine. In *B. brevis,* the first adenylation domain specifies alanine and the second adenylation domain specifies serine. Besides it contains special methylation domains such as oMT and nMT that are not found in other polyketide clusters, clearly pointing to an uncharacterized PKs encoded in this genome (Fig. 1B).

Regarding the PKs anticipated from *Bacillus*, several strains contained well described clusters for bacillaene, macrolactin and difficidin synthesis. Surprisingly, we also found variants of those, which have not been anticipated to date, even in strains of *B. amyloliquefaciens* and *B. subtilis* (Table 1 and Supplemental Table). However, prediction has to be careful here as it has been shown that small variation in the domain structure does not result in the production of different bacillaenes [31,36]. Generally, and not surprisingly *B. amyloliquefaciens* and *B. subtilis* are noted as prolific producers of PKs. Other *Bacillus* spp. encompassing PKS are *B. atrophaeus, B. mojavensis* and *Brevibacillus brevis* with clearly different PKs potential. In more detail, macrolactin variants are found in *B. amyloliquefaciens* strains such as IT-45, DC-12, UASWS BA1 and B1895 and B. *amyloliquefaciens plantarum* such as UCMB 5036, W2 and AH159-1. Bacillaene variants are found in *B. atrophaeus, B. subtilis* strains and *B. mojavensis* RRC 101. In *B. atrophaeus* and *B. mojavensis* RRC 101 variants have similar amino acids like in *B. amyloliquefaciens* FZB42 but differ in number of catalytic domains. In *B. subtilis* strains, we found variation to bacillaene as the second adenylation domain specifies glutamine, but the number of catalytic domains is identical to *B. amyloliquefaciens* FZB42. It has also to be stated that not all metabolite clusters of these species are expressed or even be functional as seen in *B. subtilis* 168 [81]. This lab strain obviously does not require its secondary metabolites anymore, very likely unlike its relatives living in plant association in nature.

Regarding the PKs anticipated from *Bacillus*, several strains contained well described clusters for bacillaene, macrolactin and difficidin synthesis. Surprisingly, we also found variants of those, which have not been anticipated to date, even in strains of *B. amyloliquefaciens* and *B. subtilis* (Table 1 and Supplemental Table). However, prediction has to be careful here as it has been shown that small variation in the domain structure does not result in the production of different bacillaenes [31,36]. Generally, and not surprisingly *B. amyloliquefaciens* and *B. subtilis* are noted as prolific producers of PKs. Other *Bacillus* spp. encompassing PKS are *B. atrophaeus, B. mojavensis* and *Brevibacillus brevis* with clearly different PKs potential. In more detail, macrolactin variants are found in *B. amyloliquefaciens* strains such as IT-45, DC-12, UASWS BA1 and B1895 and B. *amyloliquefaciens plantarum* such as UCMB 5036, W2 and AH159-1. Bacillaene variants are found in *B. atrophaeus, B. subtilis* strains and *B. mojavensis* RRC 101. In *B. atrophaeus* and *B. mojavensis* RRC 101 variants have similar amino acids like in *B. amyloliquefaciens* FZB42 but differ in number of catalytic domains. In *B. subtilis* strains, we found variation to bacillaene as the second adenylation domain specifies glutamine, but the number of catalytic domains is identical to *B. amyloliquefaciens* FZB42. It has also to be stated that not all metabolite clusters of these species are expressed or even be functional as seen in *B. subtilis* 168 [81]. This lab strain obviously does not require its secondary metabolites anymore, very likely unlike its relatives living in plant association in nature.

We also performed genome mining on Bacillales genera growing in other environments. Intriguingly, the majority of these non-plant associated bacteria do not harbor LPS and PKS. On the contrary, a large fraction of the plant-associated bacteria contained LPS and PKS (Supplemental Table, Supplemental Fig.) with both *Bacillus* and *Paenibacillus* dominating the distribution. However, bacteria such as *Ornithinibacillus* and *Salinibacillus* occuring in soil environments seem also to have the capacity to produce macrolactin-like polyketides with higher dissimilarity to the macrolactin of *B. amyloliquefaciens* FZB42.

We also performed genome mining on Bacillales genera growing in other environments. Intriguingly, the majority of these non-plant associated bacteria do not harbor LPS and PKS. On the contrary, a large fraction of the plant-associated bacteria contained LPS and PKS (Supplemental Table, Supplemental Fig.) with both *Bacillus* and *Paenibacillus* dominating the distribution. However, bacteria such as *Ornithinibacillus* and *Salinibacillus* occuring in soil environments seem also to have the capacity to produce macrolactin-like polyketides with higher dissimilarity to the macrolactin of *B. amyloliquefaciens* FZB42.

### 1.5 Conclusions and future perspectives

*Bacillus* and some related genera can be phylogenetically separated into ten distinct groups based on 16S rRNA gene sequence information [82,83]. It is intriguing that the LPS and PKS gene clusters seem to be constrained to particular species or groups (Supplemental Fig.), potentially indicating the ecological role for such gene clusters.

*Bacillus* and some related genera can be phylogenetically separated into ten distinct groups based on 16S rRNA gene sequence information [82,83]. It is intriguing that the LPS and PKS gene clusters seem to be constrained to particular species or groups (Supplemental Fig.), potentially indicating the ecological role for such gene clusters.

BLAST results can be often misleading in the prediction of metabolic capacity as part of the target gene cluster can share similarity within and between different gene clusters. Therefore, it is crucial to examine the whole architecture of a particular gene cluster to obtain precise results. With an increasing availability of genome information due to advanced and better affordable next generation sequencing, we anticipate that there will be enormous increase in the deposition of sequences in public databases derived from uncultured and less studied bacteria. Such sequence wealth can be a rich source for novel secondary metabolite production and can be explored to find novel gene clusters encoding secondary metabolites. Our results suggest that a substantial fraction of predicted LPs and PKs from the metabolomes of Bacillales are uncharacterized and their functions with regards to plant association still remains to be established and other so far neglected Bacillales with no published genomic data still remain unexplored.

## 2 Materials and methods

### 2.1 Genome sequences

NCBI accession numbers for the whole genome sequences of both characterized and uncharacterized group of isolates from selected members of the Bacillales were extracted. (Table 1, Supplemental Table). Contigs of draft genomes were extracted and saved as a fasta file.

NCBI accession numbers for the whole genome sequences of both characterized and uncharacterized group of isolates from selected members of the Bacillales were extracted. (Table 1, Supplemental Table). Contigs of draft genomes were extracted and saved as a fasta file.

### 2.2 Secondary metabolite gene cluster prediction and analysis tools

Three web based tools, antiSMASH, NaPDos, and NRPS/PKS substrate predictor tools were used for secondary metabolite gene cluster prediction and analysis. The architecture of the gene clusters were predicted using the antiSMASH program [20,21]. The catalytic domains of the predicted gene cluster are deduced using NaPDoS [22]. To analyze adenylation domains of NRPS and AT domains of PKS, NRPS/PKS substrate predictor [23] was used.

Firstly, Genbank accession numbers were given as input for antiSMASH. For draft genomes, the extracted files were uploaded to antiSMASH. The predicted secondary metabolite gene clusters from antiSMASH consisted of NRPS, PKS, hybrid PKS/NRPS, siderophore, bacteriocin and lantibiotics. The clusters responsible for biosynthesis of LPs and PKs were analyzed. Further predicted monomers were confirmed using NaPDos and NRPS/PKS substrate predictor. For accuracy, predictions from the three tools were analyzed. Regarding polyketides, the number of core catalytic domains KS, DH, KR, ACP and ER were noted. Finally, both lipopeptide and polyketide encoding gene clusters were subjected to BLAST to find the closest homologue available in the database.

### 2.3 Phylogenetic analysis of predicted LPs and type I PKs

The 16S rRNA gene sequences were downloaded from RDP [84]. These sequences were clustered at 97% identity using clustalW, and a tree was plotted using neighbor joining algorithm within MEGA6 [85]. The phylogenetic distribution of predicted LPs and PKs from genome mining is combined with the tree and visualized in iTOL2 [86].

The following are the supplementary data related to this article.

Supplemental Table Prediction of type I PKs and LPs in completed and draft genomes of the Bacillales.

Supplemental Fig Phylogenetic distribution of predicted LPs and type I PKs in Bacillales based on 16S rRNA similarities. The number of predicted LPs and type I PKs for each genome are shown in bar graph, and the colored leaf labels indicate the source of isolation (rhizosphere soil, soil and endophytes). The phylogenetic tree was visualized using iTOL2 [86].

Supplementary data to this article can be found online at http://dx.doi.org/10.1016/j.csbj.2015.03.003.

## Figures and Tables

**Table 1:** Predicted lipopeptides and type I polyketides from selected members of Bacillales.

GenBank ID	Organism	Lipopeptide*	Type I polyketide*
CP000154.1	*Paenibacillus polymyxa* E681	Polymyxin A, structure and biosynthetic gene cluster confirmed [SKChoi 2009, Catch JR 1949] L-dab-L-thr-D-dab-L-dab-L-dab-D-leu-L-thr-L-dab-L-dab-L-thr Fusaricidin C, structure and biosynthetic gene cluster confirmed [Soo-Keun Choi 2008] L-thr-D-val-L-tyr-D-thr-D-asn-D-ala Predicted tridecaptin variant D-val-D-dab-D-gly-D-ser-D-phe-L-ser-L-dab-D-dab-L-phe-L-glu-L-val-D-ile-L-val Predicted unknown heptapeptide (mal) + (pk) + D-ser-D-orn-D-phe-D-val-L-phe-L-phe-L-glu 47% identity to bacillomycin of B. *amyloliquefaciens* FZB42	Novel polyketide gly (DH = 5, KS = 12, KR = 9, cMT = 2, ACP = 14); 43% identity to known bacillaene of *B. amyloliquefaciens* FZB42
ARIL00000000.1	*Paenibacillus polymyxa* SQR-21	Polymyxin A variant L-dab-L-thr-D-dab-L-dab-L-dab-D-leu-L-leu-L-dab-L-dab-L-thr Fusaricidin C (peptide sequence is similar to E681); 93%identity to fusaricidin of P. polymyxa E681 Predicted tridecaptin variant, peptide sequence is similar to *P. polymyxa* E681 Predicted unknown heptapeptide, peptide sequence similar to *P. polymyxa* E681 Predicted decapeptide (maybe a truncated tridecaptin) D-gly-D-dab-D-gly-D-ser-D-phe-L-ser-L-dab-D-dab-L-ile-L-glu	Novel polyketide (same as above) - modular architecture is similar to *P.* *polymyxa* E681. 43% identity to bacillaene of B. *amyloliquefaciens* FZB42
ARIL00000000.1	*Paenibacillus massiliensis DSM 16942*	Novel fusaricidin variant L-thr-D-val-L-ile-D-ser-D-asn-L-ala; 49% identity to fusaricidin of *P.polymyxa* E681.	No clusters found
CP006941.1	*Paenibacillus polymyxa* CR1	Predicted heptapeptide variant (pk-nrp) + (thr-ser-ala) + (phe-gln-glu) 48% identity to bacillomycin of *B. amyloliquefaciens* FZB42	Incomplete PKS predicted
CP003235.1	*Paenibacillus* *mucilaginosus* 3016	Predicted heptapeptide variant phe + (orn-val-ile-phe-nrp-phe) 44% identity to bacillomycin of B. *amyloliquefaciens* FZB42	Incomplete PKS predicted
CP009288.1	*Paenibacillus* *durus* DSM 1735	Incomplete NRPS predicted	Paenimacrolidine (KS = 9, DH = 6, cMT = 2, KR = 6, ER = 1, ACP = 14) 40% identity to known difficidin of *B.* *amyloliquefaciens* FZB42
BAVZ00000000.1	*Paenibacillus pini* JCM 16418	Incomplete NRPS predicted	Bacillaene variant, gly; ala (KS = 14, DH = 8; KR = 8, cMT = 2, ACP = 16); also the order of domains differ; share 56% identity to bacillaene of *B.* *amyloliquifaciens* FZB42
ANAT00000000.1	*Paenibacillus* *lentimorbus* NRRL B-30488	Bacillomycin D, surfactin, plipastatin; similar to *B. amyloliquefaciens* FZB42	Bacillaene, macrolactin, difficidin; similar to *B. amyloliquefaceins* FZB42
AULE00000000.1	*Paenibacillus* *taiwanensis* DSM 18679	Paenibacterin variant (orn-val-thr-orn) + (tyr-orn-ser-ile-pro) + (pro) + (ile-ile); 69% identity with known paenibacterin of *Paenibacillus* sp. OSY-SE	Incomplete PKS predicted
ARMT00000000.1	*Paenibacillus* *fonticola* DSM 21315	Unknown heptapeptide-architecture similar to Iturin family (mal) + (pk-gly) + (orn-glu) + (lys-tyr) + (ile-val); 36% identity with known Bacillomycin of *B. amyloliquefaciens* FZB42	Incomplete PKS predicted
CP003355.1	*Paenibacillus* *larvae* DSM 25430	IturinA	Paenilamicins: A1, B1, A2, B2, -a complex NRPS/PKS hybrid lys/arg, ala, mdap, lys/orn, ser, mdap, gly (KS = 4, KR = 4, nMT = 2, ACP = 4)
CP003763.1	*Bacillus* *thuringiensis* HD- 789	Kurstakin, structure confirmed [Hathout et al. 2000] D-thr-L-gly-L-ala-L-ser-L-his-D-gln-L-gln	No clusters found
CP004069.1	*Bacillus* *thuringiensis* *serovar kurstaki* HD73	Kurstakin variant D-thr-L-ser-L-ala-L-ser-L-leu-D-nrp-L-gln 99% identity to known kurstakin of *Bacillus thuringiensis serovar kurstaki* HD-1	No clusters found
CP000560.1	*Bacillus* *amyloliquefaciens* FZB42	SurfactinA [Peypoux F 1994, Koumoutsi A 2004] L-glu-L-leu-D-leu-L-val-L-asp-D-leu-L-leu Plipastatin B [Nishikiori 1986, Koumoutsi A 2004] L-glu-D-orn-L-tyr-D-thr-L-glu-D-val-L-pro-L-gln-D-tyr-L-ile Bacillomycin D [Peypoux F 1984, Koumoutsi A 2004] L-asn-D-tyr-D-asn-L-pro-L-glu-D-ser-L-thr	Bacillaene gly; ala (KS = 14, DH = 8, KR = 9, cMT = 2, ACP = 14) Difficidin (KS = 14; DH = 9, KR = 10, cMT = 3, ER = 1, ACP = 19) Macrolactin (KS = 11, DH = 5, KR = 11, ACP = 15) [Stein, 2005; Chen et al., 2006]
JOKF00000000.1	*Bacillus* *amyloliquefaciens* *plantarum* W2	SurfactinA-similar to FZB42, Plipastatin B (similar to FZB42 but Glu instead of Gln)	Macrolactin variant (KS = 11, DH = 3, KR = 11, ACP = 15); 97% identity with known macrolactin of *B.* *amyloliquefaciens* FZB42 Difficidin variant (KS = 14, DH = 9, KR = 10, CMT = 3, ER = 0, ACP = 19); 98% identity with know difficidin of *B. amyloliquefaciens* FZB42 Bacillaene-similar to FZB42; 98% identity to bacillaene of *B.* *amyloliquefaciens* FZB42
NC_014639.1	*Bacillus* *atrophaeus* 1942	SurfactinC L-glu-L-leu-D-leu-L-val-L-asp-D-leu-L-ile; 78% identity to *B.* *amyloliquefaciens* FZB42 Plipastatin B; mycosubtilin; similar to FZB42	Bacillaene variant, similar to FZB42 in terms of specificity of A domains but (KS = 16, DH = 7, KR = 9, cMT = 2, ACP = 16); 64% identity to *B.* *amyloliquefaciens* FZB42
CM000488.1	*Bacillus subtilis* *NCIB 3610*	SurfactinA; plipastatin B; similar to FZB42	bacillaene similar to FZB42 A domains specificity gly, nrp (KS = 15, DH = 8, KR = 9, cMT = 2, ACP = 17), 64% identity to known bacillaene *of B. amyloliquefaciens* FZB42
AP008955.1	*Brevibacillus* *brevis* NBRC 100599	Incomplete NRPS predicted	Novel polyketide (KS = 14,cMT = 3, oMT = 1, nMT = 1, KR = 8, ACP = 20), A domain specificity ala, ser; 38% identity to difficidin of *B. amyloliquefaciens* *FZB42*
AEWH00000000.1	*Ornithinibacillus scapharcae* TW25	Incomplete NRPS predicted	Macrolactin like polyketide 44% identity *to B.* *amyloliquefaciens FZB42* (KS = 13, DH = 4, KR = 8, ACP = 16) Bacillaene, similar to *B.* *amyloliquefaciens* FZB42
APIS00000000.1	*Salinibacillus aidingensis* MSP4	Surfactin, plipastatin B; similar to *B. amyloliquefaciens* FZB42	Macrolactin like polyketide (KS = 12, DH = 5, KR = 6, ACP = 14) 45% idenity to bacillaene of *B. amyloliquefaciens* FZB42

* Sequence prediction using antiSMASH, NaPDos and NRPS/PKS substrate predictor tools, peptides in bold are predicted novel peptides, monomers in both bold and underline differ from described metabolites in that position (in case of polyketides they differ in number and maybe in the order of domains); monomers in underline are known variants, previously described. *B. subtilis* 3610 and *B. amyloliquefaciens* FZB42 are reported to produce similar bacillaene [Rebecca A. Butcher 2006, Chen 2009]. However, they differ in number of domains predicted. Abbreviations: mal, malonyl-CoA; pk, polyketide; dab, 2,4-diaminobutyric acid; KS, ketosynthase; DH, dehydratase; MT, methyl transferase; KR, ketoreductase; orn, ornithine, nrp, unassigned non ribosomal peptide, mdap, N-methyl-diaminopropionic acid, NRPS, non-ribosomal peptide synthetase, PKS, type 1 polyketide synthase.
