# Supplementary material for: Genome mining: Prediction of lipopeptides and polyketides from Bacillus and related Firmicutes
Source: Comput Struct Biotechnol J. 2015 Mar 24;13:192–203. doi: 10.1016/j.csbj.2015.03.003 (PMC4397504; doi:10.1016/j.csbj.2015.03.003)
Supplement: Supplemental Fig — Phylogenetic distribution of predicted LPs and type I PKs in Bacillales based on 16S rRNA similarities. The number of predicted LPs and type I PKs for each genome are shown in bar graph, and the colored leaf labels indicate the source of isolation (rhizosphere soil, soil and endophytes). The phylogenetic tree was visualized using iTOL2 [86]. [file mmc2.pdf]

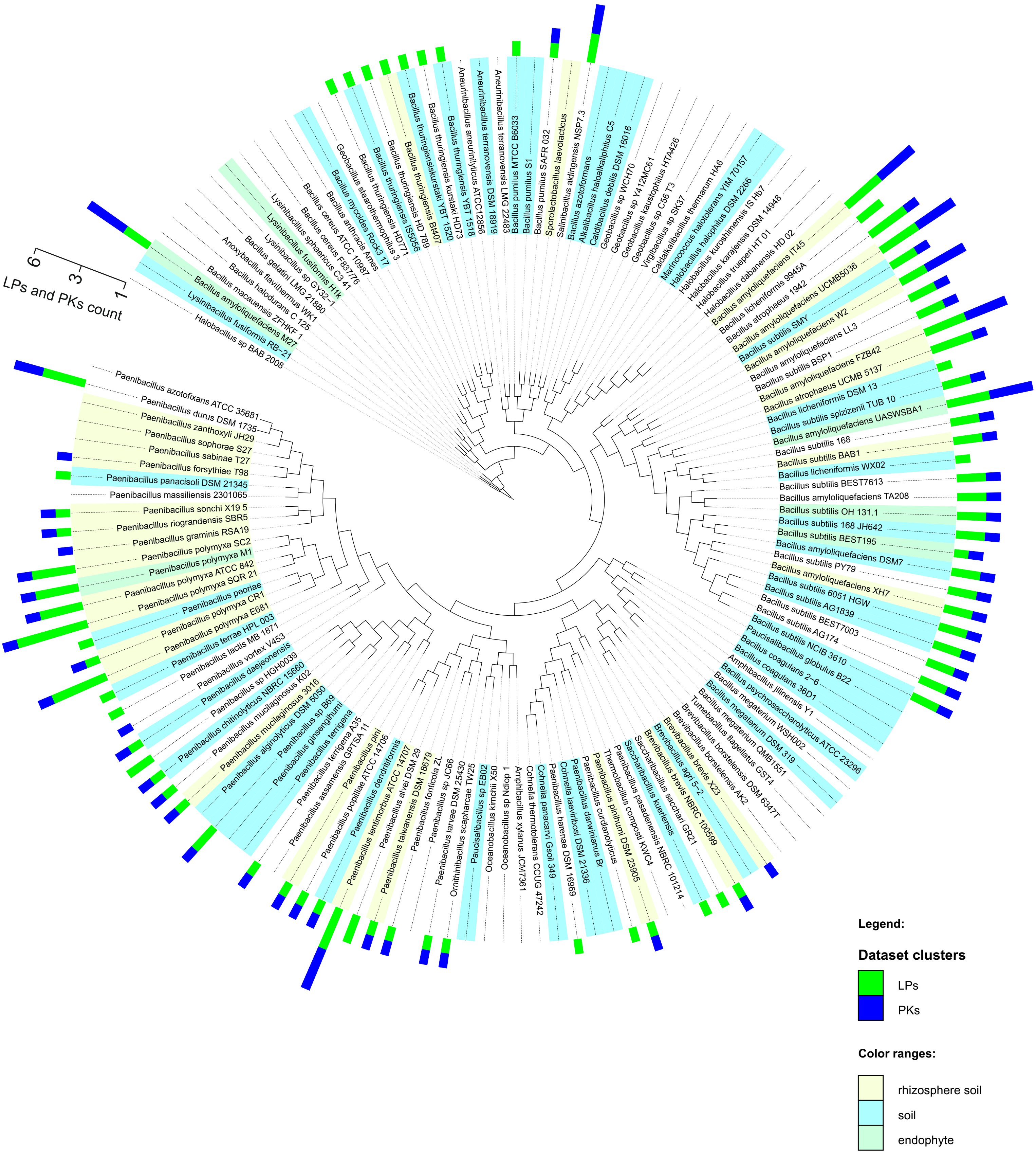
LPs and PKs count
6
3
1
Paenibacillus azotofixans ATCC 35681
Paenibacillus durus DSM 1735
Paenibacillus zanthoxyli JH29
Paenibacillus sophorae S27
Paenibacillus sabinae T27
Paenibacillus forsythiae T98
Paenibacillus panacisoli DSM 21345
Paenibacillus massiliensis 2301065
Paenibacillus sonchi X19 5
Paenibacillus riograndensis SBR5
Paenibacillus graminis RSA19
Paenibacillus polymyxa SC2
Paenibacillus polymyxa M1
Paenibacillus polymyxa ATCC 842
Paenibacillus polymyxa SQR 21
Paenibacillus peoriae
Paenibacillus polymyxa CR1
Paenibacillus polymyxa E681
Paenibacillus terrae HPL 003
Paenibacillus lactis MB 1871
Paenibacillus vortex V453
Paenibacillus daejeonensis
Paenibacillus sp HGH0039
Paenibacillus chitinolyticus NBRC 15660
Paenibacillus mucilaginosus K02
Paenibacillus mucilaginosus 3016
Paenibacillus alginolyticus DSM 5050
Paenibacillus sp B69
Paenibacillus ginsengihumi
Paenibacillus terrigena
Paenibacillus terrigena A35
Paenibacillus assamensis GPTSA 11
Paenibacillus popilliae ATCC 14706
Paenibacillus pini
Paenibacillus dendritiformis
Paenibacillus lentimorbus ATCC 14707
Paenibacillus alvei DSM 29
Paenibacillus taiwanensis DSM 18679
Paenibacillus fonticola ZL
Paenibacillus sp JC66
Paenibacillus larvae DSM 25430
Ornithinibacillus scapharcae TW25
Paucisalibacillus sp EB02
Oceanobacillus kimchii X50
Oceanobacillus sp Ndiop 1
Amphibacillus xylanus JCM7361
Cohnella thermotolerans CCUG 47242
Cohnella panacarvi Gsoil 349
Paenibacillus harenae DSM 16969
Cohnella laeviribosi DSM 21336
Paenibacillus darwinianus Br
Paenibacillus curdlanolyticus
Thermobacillus composti KWC4
Paenibacillus pinihumi DSM 23905
Saccharibacillus kuerlensis
Paenibacillus pasadenensis NBRC 101214
Saccharibacillus sacchari GR21
Brevibacillus brevis NBRC 100599
Brevibacillus agri 5-2
Brevibacillus brevis X23
Bacillus borstelensis AK2
Brevibacillus laterosporus DSM 6347T
Tumebacillus flagellatus GST4
Bacillus megaterium QMB1551
Bacillus megaterium DSM 319
Bacillus megaterium WSH002
Bacillus psychrosaccharolyticus ATCC 23296
Amphibacillus jilinensis Y1
Bacillus coagulans 2-6
Bacillus coagulans 36D1
Paucisalibacillus globulus B22
Bacillus subtilis NCIB 3610
Bacillus subtilis AG174
Bacillus subtilis BEST7003
Bacillus subtilis AG1839
Bacillus subtilis 6051 HGW
Bacillus amyloliquefaciens XH7
Bacillus subtilis PY79
Bacillus amyloliquefaciens DSM7
Bacillus subtilis BEST195
Bacillus subtilis 168 JH642
Bacillus subtilis OH 131.1
Bacillus amyloliquefaciens TA208
Bacillus subtilis BEST7613
Bacillus licheniformis WX02
Bacillus subtilis BAB1
Bacillus subtilis 168
Bacillus amyloliquefaciens UASWSBA1
Bacillus subtilis spizizenii TUB 10
Bacillus licheniformis DSM 13
Bacillus atrophaeus UCMB 5137
Bacillus amyloliquefaciens FZB42
Bacillus subtilis BSP1
Bacillus amyloliquefaciens LL3
Bacillus amyloliquefaciens W2
Bacillus subtilis SMY
Bacillus amyloliquefaciens UCMB5036
Bacillus atrophaeus 1942
Bacillus licheniformis 9945A
Bacillus amyloliquefaciens IT45
Halobacillus dabanensis HD 02
Halobacillus trueperi HT 01
Halobacillus karajensis DSM 14948
Halobacillus kuroshimensis IS Hb7
Halobacillus halophilus DSM 2266
Halobacillus halotolerans YIM 70157
Marinococcus halotolerans
Caldalkalibacillus thermarum HA6
Virgibacillus sp SK37
Geobacillus kaustophilus HTA426
Geobacillus sp C56 T3
Geobacillus sp Y412MC61
Geobacillus sp WCH70
Caldibacillus debilis DSM 16016
Alkalibacillus haloalkaliphilus C5
Bacillus azotoformans
Salinibacillus aidingensis NSP7.3
Sporolactobacillus laevolacticus
Bacillus pumilus SAFR 032
Bacillus pumilus S1
Bacillus pumilus MTCC B6033
Aneurinibacillus terranovensis LMG 22483
Aneurinibacillus terranovensis DSM 18919
Aneurinibacillus aneurinilyticus ATCC12856
Bacillus thuringiensis YBT 1518
Bacillus thuringiensis kurstaki HD73
Bacillus thuringiensis kurstaki YBT 1520
Bacillus thuringiensis BMB171
Bacillus thuringiensis HD 789
Bacillus thuringiensis IS5056
Bacillus thuringiensis HD771
Geobacillus stearothermophilus 3
Bacillus mycoides Rock3 17
Bacillus anthracis Ames
Bacillus cereus ATCC 10987
Bacillus cereus F837/76
Lysinibacillus sphaericus C3 41
Lysinibacillus fusiformis H1k
Lysinibacillus sp GY32-1
Bacillus gelatini LMG 21880
Anoxybacillus flavithermus WK1
Bacillus halodurans C 125
Bacillus macauensis ZFHKF 1
Bacillus amyloliquefaciens M27
Lysinibacillus fusiformis RB-21
Halobacillus sp BAB 2008
Legend:
Dataset clusters
LPs
PKs
Color ranges:
rhizosphere soil
soil
endophyte
